# CDK4/6 inhibition alone and in combination for non-small cell lung cancer

**DOI:** 10.18632/oncotarget.26545

**Published:** 2019-01-18

**Authors:** Jose Pacheco, Erin Schenk

**Affiliations:** University of Colorado Cancer Center, Aurora, CO, USA; University of Colorado Cancer Center, Aurora, CO, USA

**Keywords:** palbociclib, CDK4/6, NSCLC, mTOR, MEK/ERK

Increased signaling through cyclin dependent kinases 4/6 (CDK4/6) is a frequent alteration in cancer. Under appropriate growth stimuli, CDK4/6 associate with cyclin D rather than inhibitor of CDK4 (INK4) proteins. Once bound to cyclin D, CDK4/6 preferentially phosphorylate retinoblastoma protein (Rb) ultimately resulting in the cell transition from G_1_ to S phase. Multiple mechanisms increasing signaling through CDK4/6 have been described in cancer. Decreased expression of INK4 proteins is relatively common in patients with non-small cell lung cancer (NSCLC) and may occur through gene deletion, hypermethylation, or mutation. NSCLC with loss of INK4 mediated inhibition has increased signaling through CDK4/6 and is hypothesized to be more sensitive to CDK4/6 inhibitors [1]. Thus, selecting for loss of INK4 proteins (e.g., p16), may be one way to try to enrich for responders to CDK4/6 inhibition.

In this context, Gopalan et al conducted a phase 2 trial evaluating the CDK4/6 inhibitor palbociclib in patients with p16 null NSCLC who had an average of two prior treatments (range 1 – 4). There were no objective responses in the 16 evaluable patients, median progression free survival (PFS) was 3.2 months (95% CI 2.1 – 6.0) and median overall survival (OS) was 7.7 months (95% CI 4.0 – 13.5). Eight patients had stable disease (SD) (6 of whom had SD ≥ 6 months) and eight patients experienced progressive disease (PD). The available molecular analysis did not identify candidate alterations that could characterize those with improved outcomes on palbociclib [2]. The results from this trial add to the evidence that CDK4/6 inhibitors are not very effective mono-therapies in NSCLC and current attempts to enrich for responders have not yet identified a predictive marker.

In a phase I study of the CDK4/6 inhibitor abemaciclib, KRAS mutations appeared to predict disease control in heavily pretreated patients with metastatic NSCLC, with one patient experiencing a partial response [3]. Subsequently, the phase III JUNIPER trial randomized patients with advanced NSCLC harboring a KRAS mutation to abemaciclib or erlotinib (an inhibitor of epidermal growth factor receptor). Despite improved PFS with abemaciclib, there was no benefit in OS when compared to erlotinib [4]. Notably, erlotinib is no longer recommended for patients with EGFR wild-type/KRAS mutant NSCLC due to poor efficacy and is not a suitable comparator.

Squamous NSCLC has frequent disruption of p16 and alterations in cyclin D signaling, both of which may lead to increased CDK4/6 signaling [5]. As a result, two studies evaluated CDK4/6 inhibition in a predominately second line setting in patients with squamous NSCLC [5–6]. One of these trials was a randomized phase II study that did not employ a biomarker selection strategy. In this trial response rate (20.8% *vs* 2.8%) and PFS (4.2 months *vs* 2.5 months, *p* = 0.0068) were superior with standard of care docetaxel. There was also a trend towards improved OS with docetaxel in this study (*p* = 0.17). Rb expression by immunohistochemistry (not by mutational status) was evaluated in an exploratory analysis and presence/absence of Rb by immunohistochemistry did not appear to associate with outcomes on abemaciclib [5]. A second trial in squamous NSCLC studied palbociclib in those with cell cycle gene alterations. This study was stopped early as the target response rate was not reached, with only 2 of 32 patients (6%) having an objective response. Eighty-four percent of the registered patients on this study had amplification of CCND1, which is associated with hyperactivation of CDK4/6. CCND1 amplification did not associate with PFS or OS on this study. Median PFS and OS for all patients were 1.7 months and 7.2 months, respectively [6].

Taken together, these trials suggest monotherapy with CDK4/6 inhibitors is unlikely to be very effective even in NSCLC patients with clear activation of CDK4/6 signaling. Through *in vitro* drug screens, Gopalan et al identified mTOR inhibitors as effective partners that synergize with palbociclib in human lung cancer cell lines with a range of palbociclib sensitivity. Data from western blots of these cell lines suggests palbociclib treatment upregulates cyclin D1 in some cell lines and that the addition of everolimus reduces the levels of cyclin D1 in these cases. The authors hypothesize this additional layer of suppression of the CDK4/6-Rb pathway contributes to the increased inhibition of lung cancer growth seen *in vitro* with the addition of everolimus to palbociclib [2]. These findings are in agreement with previously published models of breast cancer, pancreatic cancer, and lung cancer showing CDK4/6 inhibitors upregulate cyclin D1 and that blockade of the mTOR pathway can overcome this mechanism of acquired CDK4/6 resistance [7]. Notably, Gopalan and colleagues report dual therapy with palbociclib and everolimus, while effective at restraining tumor growth *in vitro*, did not result in increased apoptotic cell death [2].

How will these findings translate into patients? They suggest concurrent CDK4/6 inhibition and mTOR inhibition may result in disease stabilization for more prolonged periods of time, which is a clinically acceptable outcome in lung cancer patients previously treated with multiple lines of therapy. However, reduction in tumor burden with CDK4/6 inhibition may require an alternative partner. Haines *et al* found increased signaling through an ERK driven mTOR pathway was associated with palbociclib resistance in lung cancer cell lines and demonstrated upstream inhibition with a MEK inhibitor plus palbociclib increased cell apoptosis. This combination of palbociclib and a MEK inhibitor, *versus* either agent alone, resulted in prolonged reduction in tumor burden in a genetically engineered mouse model of KRAS mutant NSCLC [8].

Clinical trials are ongoing in metastatic NSCLC and other solid tumors studying CDK4/6 inhibitors in combination with ERK, MEK, or mTOR inhibitors (NCT03170206, NCT02065063, NCT02857270, NCT03454035) based on these and other promising preclinical data [9]. Even if these combinations end up being more efficacious than CDK4/6 inhibitor monotherapy, a major challenge remains to identify predictive biomarkers for who will respond to such combinations.
